# Supplementary material for: A Critical View of the Application of the APEX Software (Aqueous Photochemistry of Environmentally-Occurring Xenobiotics) to Predict Photoreaction Kinetics in Surface Freshwaters
Source: Molecules. 2019 Dec 18;25(1):9. doi: 10.3390/molecules25010009 (PMC7017383; doi:10.3390/molecules25010009)
Supplement: Supplementary file 1 [file molecules-25-00009-s001.zip › 3.2.4_gcc-4.4.0.zip!/3.2.4_gcc-4.4.0/3.2.4_gcc-4.4.0/doc/octave/html/interpreter/Basic-Statistical-Functions.html]

Basic Statistical Functions - Untitled


Next: Statistical Plots,
Previous: Descriptive Statistics,
Up: Statistics


---

### 25.2 Basic Statistical Functions

Octave also supports various helpful statistical functions.

— Function File: **mahalanobis** (x, y)  
> Return the Mahalanobis' D-square distance between the multivariate
> samples x and y, which must have the same number of
> components (columns), but may have a different number of observations
> (rows).

— Function File: **center** (x)  
— Function File: **center** (x, dim)  
> If x is a vector, subtract its mean.
> If x is a matrix, do the above for each column.
> If the optional argument dim is given, perform the above
> operation along this dimension

— Function File: **studentize** (x, dim)  
> If x is a vector, subtract its mean and divide by its standard
> deviation.
>
> If x is a matrix, do the above along the first non-singleton
> dimension. If the optional argument dim is given then operate
> along this dimension.

— Function File: c = **nchoosek** (n, k)  
> Compute the binomial coefficient or all combinations of n.
> If n is a scalar then, calculate the binomial coefficient
> of n and k, defined as
>
> ```
>            /   \
>            | n |    n (n-1) (n-2) ... (n-k+1)       n!
>            |   |  = ------------------------- =  ---------
>            | k |               k!                k! (n-k)!
>            \   /
> ```
>
> If n is a vector generate all combinations of the elements
> of n, taken k at a time, one row per combination. The
> resulting c has size `[nchoosek (length (`n`),` k`),` k`]`.
>
> `nchoosek` works only for non-negative integer arguments; use
> `bincoeff` for non-integer scalar arguments and for using vector
> arguments to compute many coefficients at once.
>
>
>
> **See also:** bincoeff.

— Function File: n = **histc** (y, edges)  
— Function File: n = **histc** (y, edges, dim)  
— Function File: [n, idx] = **histc** (...)  
> Produce histogram counts.
>
> When y is a vector, the function counts the number of elements of
> y that fall in the histogram bins defined by edges. This must be
> a vector of monotonically non-decreasing values that define the edges of the
> histogram bins. So, n `(k)` contains the number of elements in
> y for which edges `(k) <=` y `<` edges `(k+1)`.
> The final element of n contains the number of elements of y
> that was equal to the last element of edges.
>
> When y is a N-dimensional array, the same operation as above is
> repeated along dimension dim. If this argument is given, the operation
> is performed along the first non-singleton dimension.
>
> If a second output argument is requested an index matrix is also returned.
> The idx matrix has same size as y. Each element of idx
> contains the index of the histogram bin in which the corresponding element
> of y was counted.
>
>
>
> **See also:** hist.

— Function File: **perms** (v)  
> Generate all permutations of v, one row per permutation. The
> result has size `factorial (`n`) *` n, where n
> is the length of v.
>
> As an example, `perms([1, 2, 3])` returns the matrix
>
> ```
>             1   2   3
>             2   1   3
>             1   3   2
>             2   3   1
>             3   1   2
>             3   2   1
> ```

— Function File: **values** (x)  
> Return the different values in a column vector, arranged in ascending
> order.
>
> As an example, `values([1, 2, 3, 1])` returns the vector
> `[1, 2, 3]`.

— Function File: [t, l\_x] = **table** (x)  
— Function File: [t, l\_x, l\_y] = **table** (x, y)  
> Create a contingency table t from data vectors. The l
> vectors are the corresponding levels.
>
> Currently, only 1- and 2-dimensional tables are supported.

— Function File: **spearman** (x, y)  
> Compute Spearman's rank correlation coefficient rho for each of
> the variables specified by the input arguments.
>
> For matrices, each row is an observation and each column a variable;
> vectors are always observations and may be row or column vectors.
>
> `spearman (`x`)` is equivalent to `spearman (`x`,` x`)`.
>
> For two data vectors x and y, Spearman's rho is the
> correlation of the ranks of x and y.
>
> If x and y are drawn from independent distributions,
> rho has zero mean and variance `1 / (n - 1)`, and is
> asymptotically normally distributed.

— Function File: **run\_count** (x, n)  
> Count the upward runs along the first non-singleton dimension of
> x of length 1, 2, ..., n-1 and greater than or equal
> to n. If the optional argument dim is given operate
> along this dimension

— Function File: **ranks** (x, dim)  
> Return the ranks of x along the first non-singleton dimension
> adjust for ties. If the optional argument dim is
> given, operate along this dimension.

— Function File: **range** (x)  
— Function File: **range** (x, dim)  
> If x is a vector, return the range, i.e., the difference
> between the maximum and the minimum, of the input data.
>
> If x is a matrix, do the above for each column of x.
>
> If the optional argument dim is supplied, work along dimension
> dim.

— Function File: **probit** (p)  
> For each component of p, return the probit (the quantile of the
> standard normal distribution) of p.

— Function File: **logit** (p)  
> For each component of p, return the logit of p defined as
>
> ```
>           logit(p) = log (p / (1-p))
> ```

— Function File: **cloglog** (x)  
> Return the complementary log-log function of x, defined as
>
> ```
>           cloglog(x) = - log (- log (x))
> ```

— Function File: **kendall** (x, y)  
> Compute Kendall's tau for each of the variables specified by
> the input arguments.
>
> For matrices, each row is an observation and each column a variable;
> vectors are always observations and may be row or column vectors.
>
> `kendall (`x`)` is equivalent to `kendall (`x`,` x`)`.
>
> For two data vectors x, y of common length n,
> Kendall's tau is the correlation of the signs of all rank
> differences of x and y; i.e., if both x and
> y have distinct entries, then
>
> ```
>                    1
>           tau = -------   SUM sign (q(i) - q(j)) * sign (r(i) - r(j))
>                 n (n-1)   i,j
> ```
>
> in which the
> q(i) and r(i)
> are the ranks of
> x and y, respectively.
>
> If x and y are drawn from independent distributions,
> Kendall's tau is asymptotically normal with mean 0 and variance
> `(2 * (2`n`+5)) / (9 *` n `* (`n`-1))`.

— Function File: **iqr** (x, dim)  
> If x is a vector, return the interquartile range, i.e., the
> difference between the upper and lower quartile, of the input data.
>
> If x is a matrix, do the above for first non-singleton
> dimension of x. If the option dim argument is given,
> then operate along this dimension.

— Function File: **cut** (x, breaks)  
> Create categorical data out of numerical or continuous data by
> cutting into intervals.
>
> If breaks is a scalar, the data is cut into that many
> equal-width intervals. If breaks is a vector of break points,
> the category has `length (`breaks`) - 1` groups.
>
> The returned value is a vector of the same size as x telling
> which group each point in x belongs to. Groups are labelled
> from 1 to the number of groups; points outside the range of
> breaks are labelled by `NaN`.
